# Supplementary figures and images for: The Pleiades are a cluster of fungal effectors that inhibit host defenses
Source: PLoS Pathog. 2021 Jun 24;17(6):e1009641. doi: 10.1371/journal.ppat.1009641 (PMC8224859; doi:10.1371/journal.ppat.1009641)

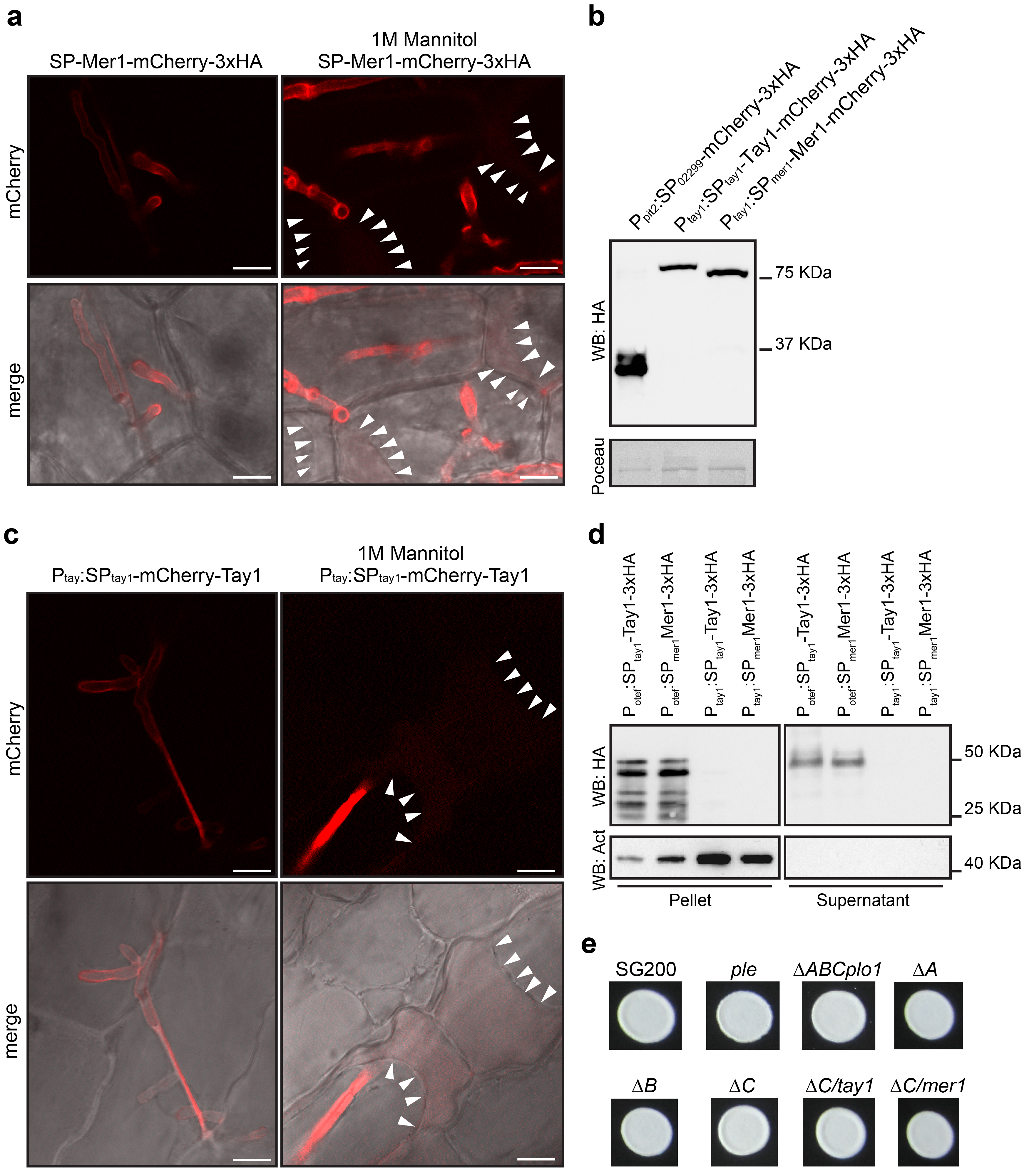

Supplement: S1 Fig — a. Secretion of Mer1 during maize infection. Left: Plants infected with the U. maydis strain SG200ΔC carrying the construct Ptay1:SPMer1-Mer1-mCherry-3xHA in the ip locus show accumulation of the mCherry signal at the hyphal edges and tips. Right: plants infected with the same strain and plasmolyzed with 1 M mannitol show accumulation of the mCherry signal in the hyphae as well as the apoplastic space. Arrowheads: plasma membranes of plasmolyzed maize cells. Upper panels: mCherry fluorescence, lower panels: bright field-mCherry merge. Scale bar = 10 μm. Pictures were taken 4 dpi. b. The expression and integrity of Ptay1:SPTay1-Tay1-mCherry-3xHA and Ptay1:SPMer1-Mer1-mCherry-3xHA during maize infection was monitored by western blot with α-HA antibodies. Secreted mCherry-3xHA is included for size reference. Tissue was collected 4dpi. c. Secretion of Tay1 during maize infection. Left: Plants infected with the U. maydis strain SG200ΔC carrying the construct Ptay1:SPTay1-mCherry-Tay1 in the ip locus show accumulation of the mCherry signal at the hyphal edges, tips and cell-to-cell crosses. Right: plants infected with the same strain and plasmolyzed with 1 M mannitol show accumulation of the mCherry signal in the hyphae as well as the apoplastic space. Arrowheads: plasma membranes of plasmolyzed maize cells. Upper panels: mCherry fluorescence, lower panels: bright field-mCherry merge. Scale bar = 10 μm. Pictures were taken 3–4 dpi. d. Secretion of Tay1 and Mer1 in axenic culture. Constructs harboring SPTay1Tay1-3×HA or SPMer1Mer1-3×HA were expressed in the strain AB33 under the tay1 or otef promoter. Total proteins were extracted from the pellet and secreted proteins were precipitated from the culture supernatant. The extracts were subjected to western blot with α HA or α Actin antibodies. Tay1-3×HA and Mer1-3×HA could be detected in the pellet and supernatant fractions only when the expression was driven by the strong synthetic otef promoter. Actin could only be detected [file ppat.1009641.s001.tif]

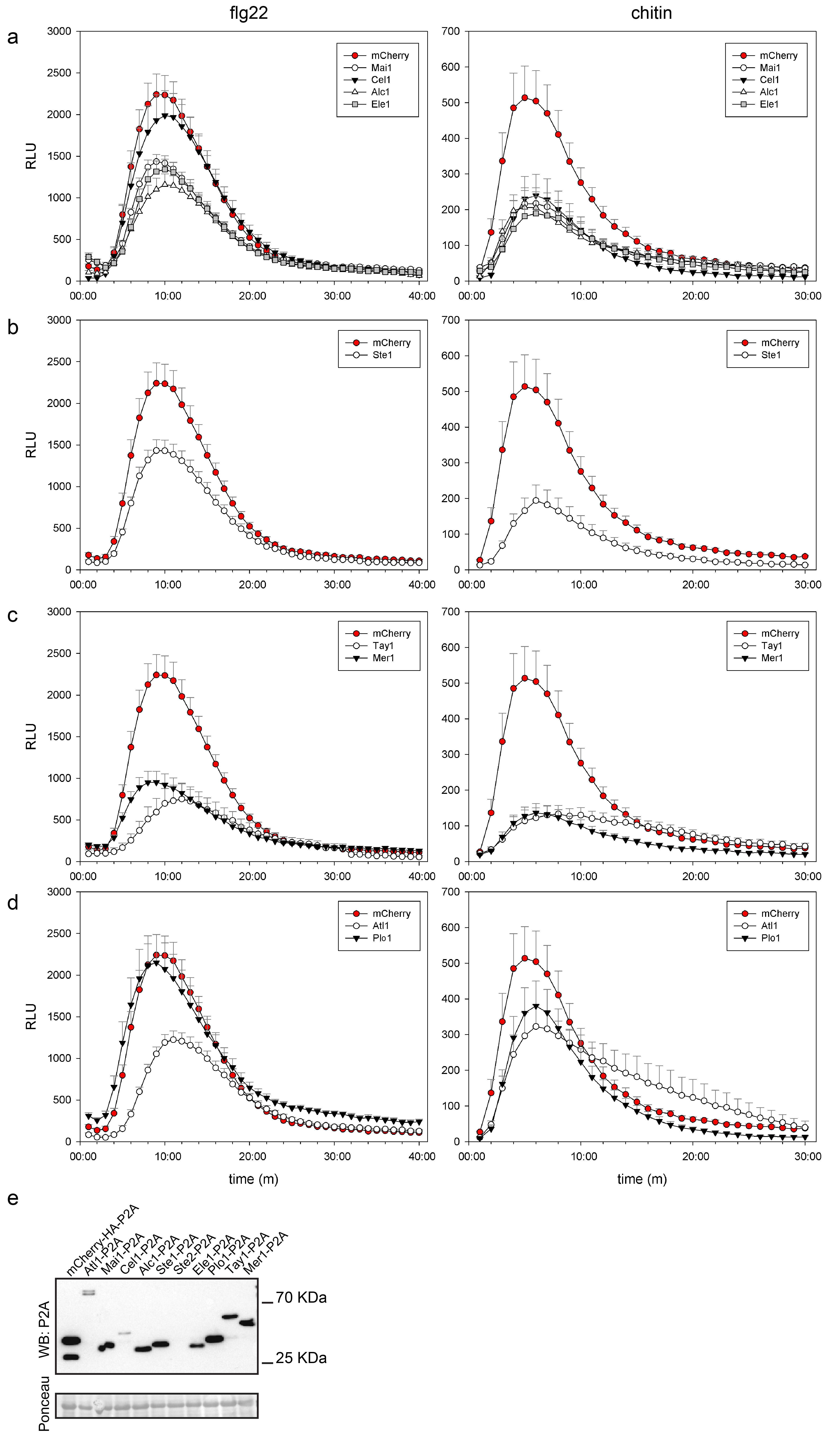

Supplement: S2 Fig — PAMP-triggered ROS burst curves corresponding to Fig 2B. Left: flg22, right: chitin. a. Curves corresponding to proteins from family A. b. Curves corresponding to proteins from family B. c. Curves corresponding to proteins from family C. d. Curves corresponding to Atl1 and Ple1. Data is mean ± SEM, n = 15 (flg22) or 12 (chitin). Only the upper error bar is shown for clarity. All the measurements were done simultaneously; curves were split according to protein family only for clarity. e. The expression and integrity of the Pleiades was monitored in N. benthamiana by western blot with α-P2A antibodies. mCherry was included as reference. Notice that Ste2 was not detected. (TIF) [file ppat.1009641.s002.tif]

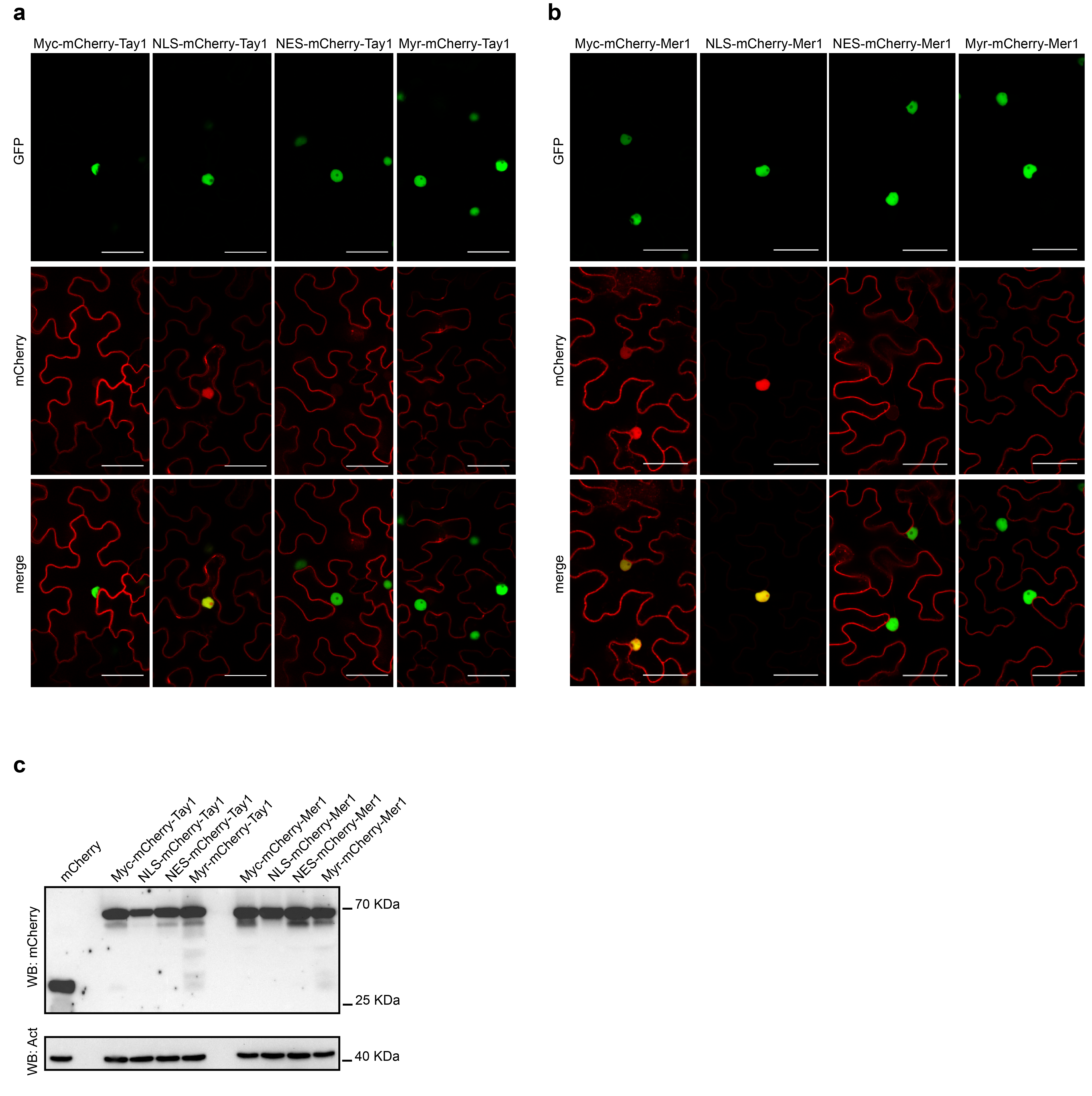

Supplement: S3 Fig — GFP-NLS was co-expressed as a nuclear marker. a. Myc-mCherry-Tay128-398, NES-mCherry-Tay128-398 and Myr-mCherry-Tay128-398 localize largely outside of the nucleus, whereas NLS-mCherry-Tay128-398 localizes to the nucleus and cytoplasm. b. Myc-mCherry-Mer123-341 localizes to the nucleus and cytoplasm, NLS-mCherry-Mer123-341 localizes to nucleus, whereas NES-mCherry-Mer123-341 and Myr-mCherry-Mer123-341 localize to the cytoplasm. All scale bars = 50 μm. c. The expression of Tay1 and Mer1 mCherry fusions used in a and b was monitored by western blot with α-mCherry antibodies. α Actin western blot was used as loading control. (TIF) [file ppat.1009641.s003.tif]

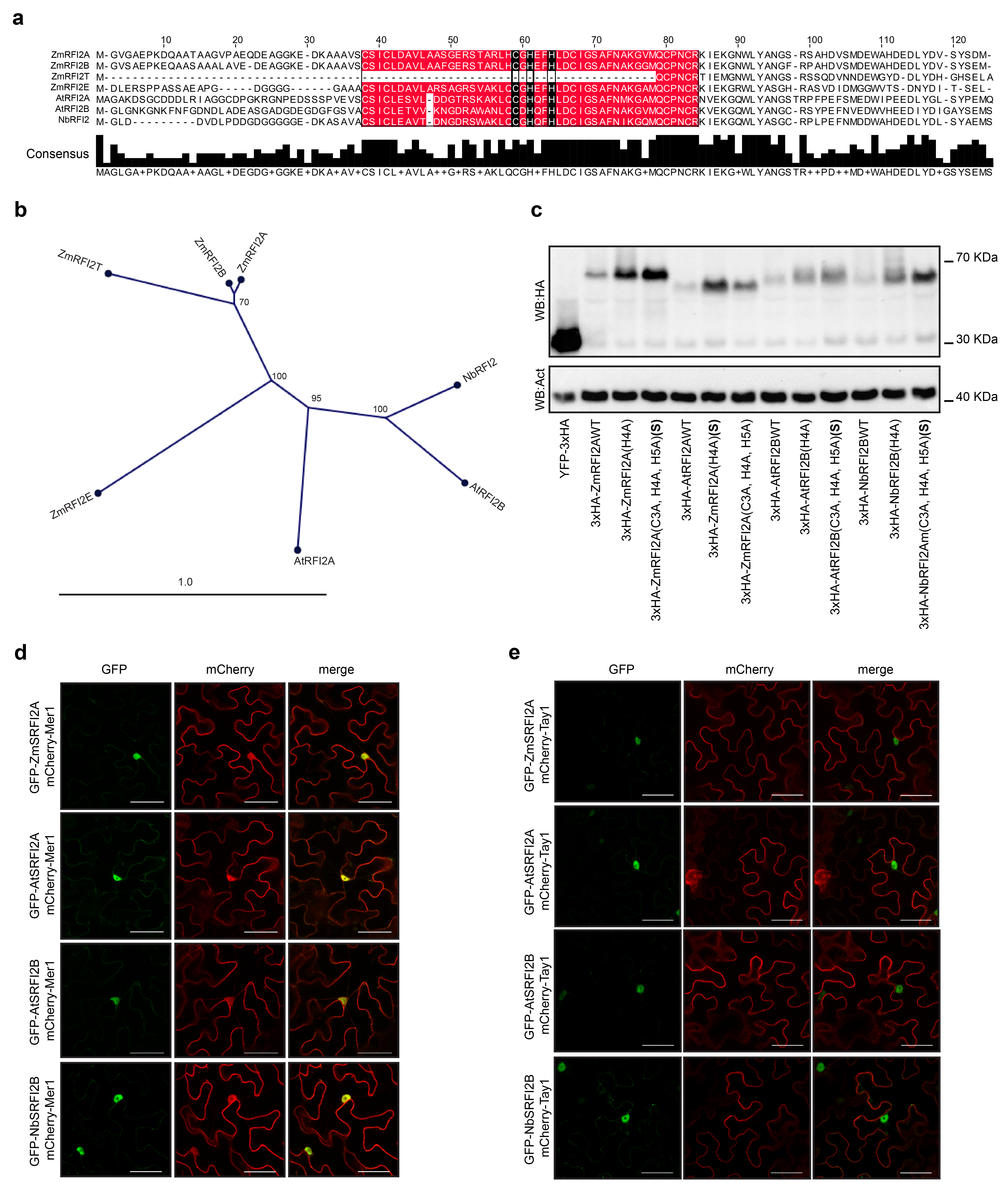

Supplement: S4 Fig — a. Protein alignment of the N-terminus of RFI2 family members shown in Fig 4A. The residues in red background mark the RING domain. Within the RING domain, Zn-coordinating residues 3, 4 and 5 are marked in black background. b. Maximum likelihood, un-rooted phylogenetic tree of the RFI2 homologs shown in a. Branch length represents genetic distance according to Kimura 2-parameter. c. Mutations of the Zn-coordinating residues stabilizes RFI2s. α-HA western blot showing the expression of WT, single or triple alanine substitutions of the residues shaded in black in part a. For each protein, the most stable mutant is marked with “S”. YFP-3xHA is shown for comparative reasons. α Actin western blot was used as loading control. c. d. Co-localization of Mer1 and Tay1 with different members of the RFI2 family in the epidermis of N. benthamiana. mCherry-Mer123-341 co-localizes with GFP-ZmSRFI2A, GFP-AtSRFI2A, GFP-AtSRFI2B and GFP-NbSRFI2 at the plant nucleus (d), whereas mCherry-Tay128-398 does not (e). Scale bar = 50 μm. (TIF) [file ppat.1009641.s004.tif]

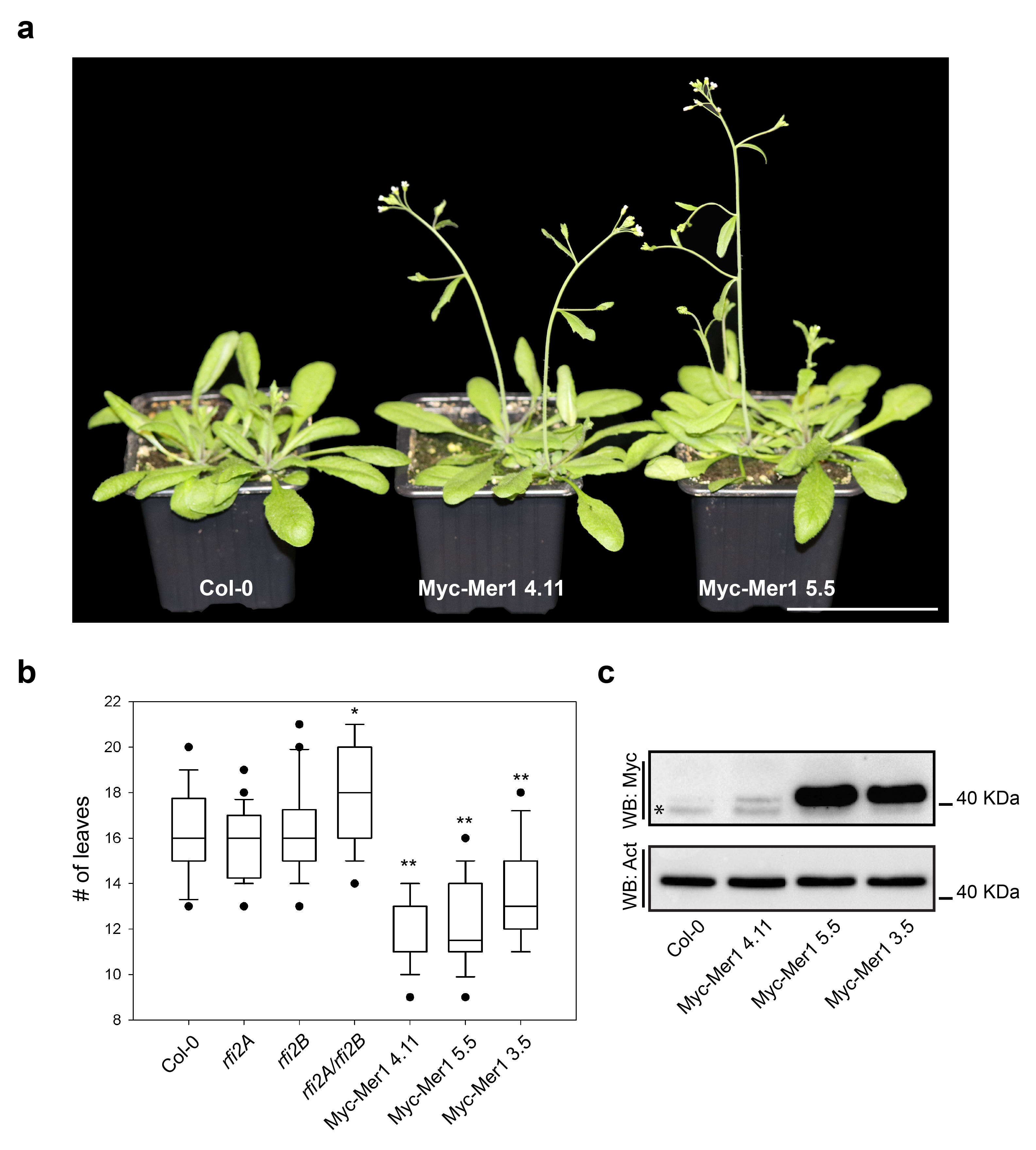

Supplement: S5 Fig — a. Flowering of Col-0 and two independent Myc-Mer123-341 lines 29 days after planting. Plants were grown in long day conditions (16h l/8h d). Scale bar = 6 cm. b. Quantification of flowering time, assessed as number of leaves at flowering day, is shown as box plots. 35S:Myc-Mer123-341 plants show early flowering whereas the rfi2A, rfi2B and rfi2A/rfi2Bknockouts in the Col-0 background do not. One representative experiment is shown, n = 30 plants. Significant differences between lines were analyzed by ANOVA, Tukeys (* p<0.05, ** p<0.01). c. Expression of Mer123-341 in A. thaliana. Expression was assessed by western blot with α-Myc antibodies, α-Actin was used as loading control. “*” unspecific band. (TIF) [file ppat.1009641.s005.tif]

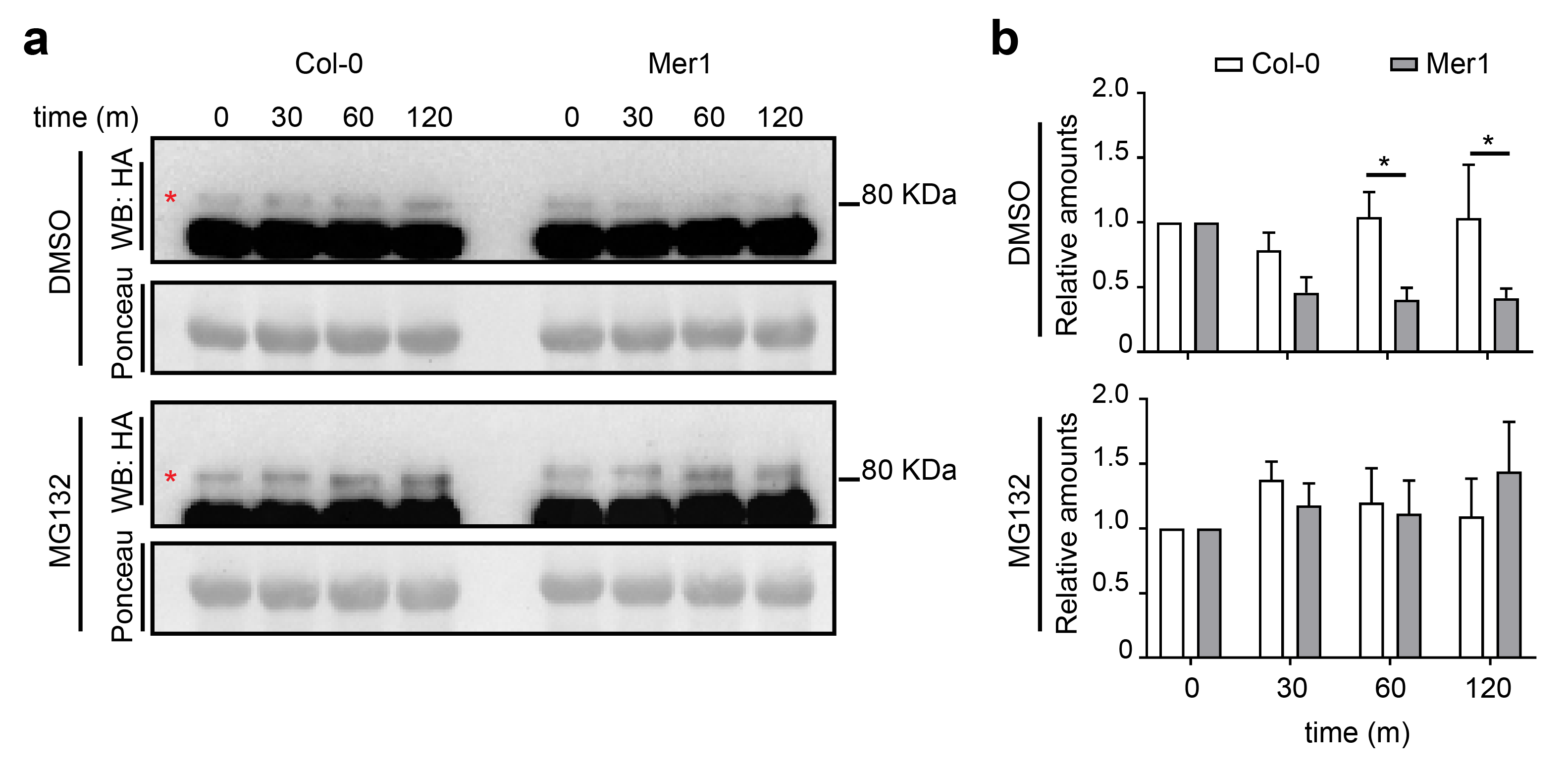

Supplement: S6 Fig — a. Cell free degradation assay in A. thaliana. MBP-HA-AtRFI2A was purified from E. coli, incubated in Col-0 or 35S:Myc-Mer123-341 crude extracts and its stability was monitored over time by western blot with α-HA antibodies. (*) indicates the full-length protein. Ponceau staining was used as loading control. MG132 was used to assess proteasomal activity on the stability of the recombinant protein. b. Protein quantification over time. Data, mean ± SEM, is a pool of four independent experiments. Significant differences were analyzed by Two way- ANOVA with Benjamini-Hochberg correction for multiple comparisons (* p<0.05). (TIF) [file ppat.1009641.s006.tif]
