# Supplementary material for: The Pleiades are a cluster of fungal effectors that inhibit host defenses
Source: PLoS Pathog. 2021 Jun 24;17(6):e1009641. doi: 10.1371/journal.ppat.1009641 (PMC8224859; doi:10.1371/journal.ppat.1009641)
Supplement: S1 Table — (DOCX) [file ppat.1009641.s007.docx]

**S1 Table** Homology within each of the Pleiades families in *U. maydis*.

|  | | Family A | | | | Family B | | Family C | |
| --- | --- | --- | --- | --- | --- | --- | --- | --- | --- |
|  |  | Mai1 | Cel1 | Alc1 | Ele1 | Ste1 | Ste2 | Tay1 | Mer1 |
| Family A | Mai1 | --- | --- | --- |  | --- | --- | --- | --- |
|  | Cel1 | 27.27 | --- | --- | --- | --- | --- | --- | --- |
|  | Alc1 | 24.68 | 25.28 | --- | --- | --- | --- | --- | --- |
|  | Ele1 | 71.48 | 27.48 | 24.32 | --- | --- | --- | --- | --- |
| Family B | Ste1 | --- | --- | --- | --- | --- | --- | --- | --- |
|  | Ste2 | --- | --- | --- | --- | 28.91 | --- | --- | --- |
| Family C | Tay1 | --- | --- | --- | --- | --- | --- | --- | --- |
|  | Mer1 | --- | --- | --- | --- | --- | --- | 30.86 | --- |

Homology is shown as % of identity.
