# Supplementary material for: The Pleiades are a cluster of fungal effectors that inhibit host defenses
Source: PLoS Pathog. 2021 Jun 24;17(6):e1009641. doi: 10.1371/journal.ppat.1009641 (PMC8224859; doi:10.1371/journal.ppat.1009641)
Supplement: S2 Table — (DOCX) [file ppat.1009641.s008.docx]

**S2 Table**

Conservation of the Pleiades proteins across different smut species.

| Host | *Zea mays* | | | *Saccharum spp.* | *Hordeum vulgare* | *Brachypodium spp.* | *Persicaria spp.* |
| --- | --- | --- | --- | --- | --- | --- | --- |
| Smut species | *U. maydis* | | *S. reilianum* | *S. scitamineum* | *U. hordei* | *U. bromivora* | *M. pennsylvanicum* |
| Ortholog Group | UMAG_03743 * | --- | sr11224 (58) | SPSC_05102 (59) | UHOR_15312 (49) | UBRO_15312 (51) | BN887_00222 (50) |
|  | UMAG_03744 | Atl1 | --- | --- | --- | --- | --- |
|  | UMAG_03745 | Mai1 | sr11231 (31) | SPSC_05105 (25) | --- | --- | --- |
|  | UMAG_03746 | Cel1 | sr20008 (42) | SPSC_05106 (36) | UHOR_14479 (30) | UBRO_14479 (34) | BN887_00270 (27) |
|  | UMAG_03747 | Alc1 | sr11233 (30) | SPSC_05108 (37) | UHOR_14481 (33) | UBRO_14481 (35) | --- |
|  | UMAG_03748 | Ste1 | --- | SPSC_05109 (33) | --- | UBRO_20303 (28) | --- |
|  | UMAG_03749 | Ste2 | sr11234.2 (41) | SPSC_05110 (42) | UHOR_07107 (29) | UBRO_07107 (30) | --- |
|  | UMAG_03750 | Ele1 | sr11231 (32) | SPSC_05105 (33) | --- | --- | --- |
|  | UMAG_03751 | Plo1 | sr11236.2 (48) | SPSC_05112 (51) | --- | --- | --- |
|  | UMAG_03752 | Tay1 | sr11237 (37) | SPSC_05114 (36) | UHOR_14482 (28) | UBRO_20121 (30) | BN887_00268 (28) |
|  |  |  |  |  | UHOR_07319 (32) | UBRO_08139 (31) | BN887_03415 (34) |
|  |  |  |  |  |  |  | BN887_00267 (29) |
|  | UMAG_03753 | Mer1 | sr11240 (32) | SPSC_05116 (35) | --- | --- | --- |
|  | UMAG_03754 * | --- | sr11241 (59) | SPSC_05117 (61) | UHOR_05718 (50) | UBRO_05718 (47) | BN887_00266 (53) |

In brackets: % of identity
“---”: Absence of the ortholog
“*”: Genes neighbouring the Pleiades cluster (which do not contain a predicted secretion signal).
