# Supplementary material for: The Pleiades are a cluster of fungal effectors that inhibit host defenses
Source: PLoS Pathog. 2021 Jun 24;17(6):e1009641. doi: 10.1371/journal.ppat.1009641 (PMC8224859; doi:10.1371/journal.ppat.1009641)
Supplement: S3 Table — (DOCX) [file ppat.1009641.s009.docx]

**S3 Table**

Prediction of signal peptides across the *U. maydis* Pleiades.

| locus | UMAG 03743 | UMAG 03744 | UMAG 03745 | | UMAG 03746 | UMAG 03747 | UMAG 03748 | UMAG 03749 | UMAG 03750 | UMAG 03751 | UMAG 03752 | UMAG 03753 | UMAG 03754 |
| --- | --- | --- | --- | --- | --- | --- | --- | --- | --- | --- | --- | --- | --- |
| name | --- | Atl1 | Mai1 | | Cel1 | Alc1 | Ste1 | Ste2 | Ele1 | Plo1 | Tay1 | Mer1 | --- |
| Signal peptide (aa) | --- | 1-33 | | 1-18 | 1-26 | 1-26 | 1-16 | 1-21 | 1-18 | 1-31 | 1-27 | 1-22 | *---* |
|  |  |  |  |  |  |  |  |  |  |  |  |  |  |
|  |  |  |  |  |  |  |  |  |  |  |  |  |  |
| Score | 0.0116 | 0.9834 | 0.9967 | | 0.9015 | 0.9015 | 0.991 | 0.998 | 0.9967 | 0.9489 | 0.9815 | 0.98 | 0.0006 |

Signal peptides were predicted with SignalP-5.0.
The signal peptides are shown in amino acids.
Score: likelihood of the prediction (1 being the highest).
“---”: Lack of a secretion signal.
