# Supplementary material for: The Pleiades are a cluster of fungal effectors that inhibit host defenses
Source: PLoS Pathog. 2021 Jun 24;17(6):e1009641. doi: 10.1371/journal.ppat.1009641 (PMC8224859; doi:10.1371/journal.ppat.1009641)
Supplement: Attachment — Submitted filename: 02 Response to reviewers.docx [file ppat.1009641.s011.docx]

**Response to the reviewers (in blue)**

Reviewer #1: I only have one major issue, which concerns the final interpretation of how Mer1 functions, which is primarily based on the in vitro experiments on auto-ubiquitination of the E3 ligase and its degradation. The authors conclude by saying that the data “indicate” and they leave no doubt that Mer1 inhibits RFI2 (l. 333-336). There could certainly be other unfound mechanisms explaining how Mer1 works. Therefore, I should like to see mechanistic studies of this in planta. One could test whether the ROS suppressing function of Mer1 is dependent on AtRFI2A in Arabidopsis. This could easily be addressed by crossing a Mer1 overexpressing line from Fig. 5a with the rfi2A mutant. The resulting Mer1 OE rfi2A line should have a ROS response indistinguishable from the parents. Even though such a result only would show that Mer1 affects the same pathway as RFI2, it would provide further support for the authors interpretation.

In our manuscript we do not exclude additional functions of Mer1 but our cumulative data indeed “indicate” a ROS-burst suppressive activity of Mer1 via RFI2 deactivation.

This are

- interaction between Mer1 and RFI2 orthologs (Y2H and Co-IP)(Fig. 4)
- Colocalisation of Mer1 with RFI2 in maize cells (Fig.4) *N. benthamiana* (S4d)
- phenotypic compliance of Mer1 OE and rfi2 deletion plants in respect to PAMP-triggered ROS responses (Fig. 5) and also early flowering phenotypes of Mer1 OE which correlates with previously published data on rfi2 mutants in *A. thaliana* accession Wassilewskija. (FigS5)
- *In vitro* promotion of autoubiquitination of RFI2 in the presence of Mer1 (Fig. 5)
- RFI2 destabilization in a cell-free degradation assay in the presence of Mer1 (FigS6)

Additionally, the positive role of RFI2 in plant immunity has been shown in rice and is mentioned in the manuscript (reference 36). We agree that the proposed epistasis-experiment suggested by reviewer 1 would be in principal interesting but it would on one hand mean crossing and backcrossing over several generations without providing more than a possible further supporting point in the experimental row listed above. More importantly, it is beside the point as we do not exclude additional targets for Mer1 as an effector and we added a sentence in the corresponding part to stress this point. Lines 333-334 (in the no track changes version).

Reviewer #2: I have the concerns as the following:

The authors state that pleiades cluster encodes ten proteins, why the authors only choose UMAG_03752 and UMAG_03753 for further investigation.

As stated in the text (page 9, lines 216-218 in the no track changes version), we focused on the strongest ROS-burst suppressors which at the same time lead to a detectable virulence defect upon deletion. Studying effector proteins means a *de novo* analysis of so far unknown proteins without homology to so far studied proteins. Identifying for eight of them a functional relevance in ROS burst suppression during biotrophy and studying two of them in more molecular detail down to the putative mechanism (in the case of Mer1) is in our eyes a publishable achievement.

The protein expression of mCherry-Mer1 and SP-mCherry-Mer1 in Fig. 1b should support with WB results.

We now performed the Westernblot of Mer1-mCherry and Tay1-mCherry to demonstrate full integrity of the proteins. As control we included free mCherry secreted by the fungus. The data is shown in the new version of Figure S2.

It’s better to use yeast invertase secretion assay to validate the predicted signal peptide of UMAG_03752 and UMAG_03753.

The secretion is demonstrated by the use of the endogenous fungal system in maize by microscopy and in axenic culture by a supernatant precipitation assay followed by western blot including respective controls (Fig. 1 and Fig. S1). We think that the heterologous yeast invertase assay should not provide more reliable results than the native system.

The authors expressed ten proteins in N. benthamiana and tested their ability to suppress PAMP-triggered ROS production, the authors should check whether these proteins are stable by using WB.

The respective proof of expression is now added as supplemental file Fig.S2.

Is there any way that we could understand the function of Tay1 and Mer1 in planta? e.g. Could they promote infection ?

As the cluster as well as the sub-cluster deletion show no growth defect (FigS1) but a significant virulence defect (Fig.1), the proof that Tay1 and Mer1 both promote infection in the native system maize is shown.

At least, one of the either BIFC or Pull down data is required to confirm the interactions between effectors and RFI2 homologs.

Fig. 4 shows Mer1 Interaction with RFI2 orthologs by Y2H and CoIP

The authors state that single knockouts (rfi2A and rfi2B) and the double knock out (rfi2A/rfi2B) plants showed a reduced ROS burst. Whether these mutants display clear phenotypes in real infection?

We used the *Arabidopsis* system, due to its amenability to genetic manipulation, to gain insights into the mechanism of action of Mer1 and its targets. We showed in the maize-*Ustilago* system that the double knock out tay1/mer1 has a detectable virulence defect and that it can be complemented by mer1 and therefore linked ZmRFI2s to host defense in this system. In addition, an RFI2 homolog in rice has also been shown to have reduced resistance to *M. oryzae*. Therefore, we did not further study the mutant phenotypes of rfi2A and B in *Arabidopsis.*

Reviewer #3: In Figure 1, the authors showed the secretion of effector protein by fusion with mCherry protein and microscopy. Secretion of effector protein is also supported by the data in Fig S1, which shows the detection of full-length protein in culture supernatant. However, it is not clear whether the secreted effector is full-length in planta. If the effector proteins lack cysteine residues providing stable protein folding, the effector might be unstable in apoplastic space. Immunoprecipitation and western blot of effector proteins from infected leaf tissues would be helpful to clarify this point.

We now performed a westernblot from infected tissue for the mCherry-fused secreted versions of Tay1 and Mer1 providing evidence for full integrity of the proteins. As control secreted mCherry was used. Fig S1.

In Figure 2, the authors showed DAB staining in the leaves infected with U. maydis wild type and cluster mutant to claim ROS accumulation in apoplastic space. Indeed, the hyphae of cluster mutant shows staining along with hyphae, while the wild type does not show it at all. However, the staining pattern looks a little bit weird for me, so I am not sure whether ROS actually accumulated in apoplastic space. To my knowledge, ROS accumulation pattern upon fungal infection is generally seen as accumulation in entire plant cell or as halo at infection sites. When I enlarged a picture, I could see some speckles of DAB staining inside fungal hyphae, which makes me doubt that ROS production actually comes from fungal side (by stresses due to plant defenses, for example). One possible experiment to clarify this point might be a gene silencing of plant genes related to ROS production (e.g. NADPH oxidase) in infected leaf tissues and microscopy to observe whether DAB staining is present or not. Another is to monitor the expression of plant genes related to ROS production upon cluster mutant infection (this would be much easier to imply that ROS production is from plant side).

PAMP-triggered ROS-burst occurs in the apoplastic space. As the plant cells do not undergo cell-death and infection by the ∆*ple* mutant still occurs (although to a lower degree) we were not surprised to see DAB-staining mainly around the hyphae. Beside this, the evidence that many Pleiades effectors are involved in ROS-suppression is mainly based on *in planta* expression assays of the Pleiades effectors followed by PAMP-triggered ROS-burst assays. As in this assay the fungus is not present, we are solely looking at the effect of the respective fungal protein when expressed inside the plant cell without signal peptide. Integrating further the secretion data of Fig. 1 and FigS1, all indications are that the Pleiades are secreted and translocated into the plant cell to suppress ROS-burst.

The suggested experiment of silencing plant genes related to ROS production (e.g. NADPH oxidase) in infected leaf tissues and microscopy to observe whether DAB staining is present or not is hardly to manage as silencing in maize is rather patchy and far from being systemic, as we learned. The alternative suggestion, to relate expression of plant genes related to ROS production to cluster mutant infections would likely work but would not provide the requested proof as also in an attenuated strain with reduced ability to infect maize. Enhanced host defense responses can be expected due to pathogen recognition and lack of corresponding effector-mediated host immunity suppression. Because of the reasons explained above, we refrained from doing the suggested experiment. We can confirm and show (FigS1) that the ∆*ple* mutant strain behaves normal on plate and has no impairment in the formation of filaments or other pleiotropic phenotypes (FigS1e).

Last and most major concern is the biological relevance of RFI2 and U. maydis infection in maize. It is very nice result that Mer1 promotes auto-ubiquitination of RFI2 and RFI2 contributes to ROS production in Arabidopsis. However, I miss that the authors do not show whether RFI2 actually contributes to ROS production and is involved in plant immunity upon U. maydis infection in maize. Straightforward experiment to demonstrate could be gene silencing of RFI2 in maize and evaluate fungal virulence to such gene-silenced maize plants, since FoMV-based virus gene silencing should be available in maize.

We agree with the reviewer that this experiment would have been an asset. Unfortunately, though we are limited with our system. As mentioned above, silencing in maize is not as systemic as one would wish. In combination with the rather moderate virulence defect of the sub-cluster c mutant such an evaluation of the direct relevance of RFI2 orthologs in maize will fail. One technical problem would be e.g. that silencing needs to be induced several days before infection in over hundred maize plants per strain, but infection is done 7 days post germination. For *U. maydis* strains with a penetration defect, the suggested experiment might work but not for strains with a moderate virulence phenotype.

The role of RFI2 in plant immunity was nevertheless shown in rice and is mentioned in our manuscript (Ref.36). Taken together, with the data in *A. thaliana* presented in the manuscript, it strongly suggests a conserved role as a positive player in PTI responses. That all biotrophic pathogens (comprising *U. maydis*) need to suppress, in one or another way, PTI-responses is consensus in the field of plant pathology and supported by innumerable studies.

**Part III – Minor Issues: Editorial and Data Presentation Modifications**
Please use this section for editorial suggestions as well as relatively minor modifications of existing data that would enhance clarity.

Reviewer #1: Minor issues:
• Please discuss how the E3 degradation may prevent ROS production.

We discussed this now in our resubmitted manuscript. Lines 359-361(in the no track changes version).

• Care should be taken to distinguish genes and proteins. Examples: In l. 33, “a cluster of ten effectors” should be a “cluster of ten effector genes”. Same in l. 346.

Thanks, we carefully went through the manuscript to correct for it.

• The Materials and Methods could do with a careful make-over. Many mistakes appear in the text, eg. regarding when to spell numbers or use numerals

We apologize, we revisited the materials and methods part to carefully correct for mistakes.

Reviewer #2: It’s better to use a well-known nuclear protein as a nuclear marker instead of GFP-NLS.

GFP -NLS nicely localizes in the plant nucleus, therefore we cannot understand why it should not be a suitable organelle marker. As it is not interfering in the plant metabolism, we believe that it is even more suitable than the overexpression of any native nuclear protein which could lead to unwanted side-effects inside the cell.

Also, a bright image is required to show the cells in fig. 3b.

The cellular localization of Tay1 and Mer1 fusing with Myc/Myr/NES/NLS-mCherry should provide. Also, WB of these proteins should be provided.

We updated figure 3b (and Fig 4c, to maintain the same criteria) to contain the bright field channel. Corresponding subcellular localization of the effector constructs as well as western blot are shown in FigS3.

The authors state that BD-Tay did not lead to the identification of any reproducible interactors. But why BD-Tay was able to interact with some of the RFI2 homologs in Y2H? Please explain!

Taygeta did not identify, by classical Y2H screening, a specific interaction partner. In case of the targeted Y2H with RFI2, we observe some interaction as shown (Fig 3a) but it could not be confirmed by Co-IP (Fig 3b). In addition, Tay1 localization and its ROS-burst suppressive activity solely outside of the nucleus do not support the Tay1-RFI2 as a biologically relevant interaction. We assume, based on the data mentioned, that the observed weak interaction between Tay1 and RFI1 is reflecting its sequence similarity to Mer1 and due to the forced nuclear localization in the Y2H system, this allows an interaction with some RFI homologs.

Although I am not a native speaker, I think the manuscript need to be carefully polished.

The manuscript was proof-read by a native speaker to improve its English quality.

----------------------------------------------------------------------------------------------------

We thank all three reviewers for their input and time invested in improving our manuscript.
