# Supplementary material for: The Pleiades are a cluster of fungal effectors that inhibit host defenses
Source: PLoS Pathog. 2021 Jun 24;17(6):e1009641. doi: 10.1371/journal.ppat.1009641 (PMC8224859; doi:10.1371/journal.ppat.1009641)
Supplement: Attachment — Submitted filename: 02 Answer to reviewers_AD.docx [file ppat.1009641.s012.docx]

**Part III – Minor Issues: Editorial and Data Presentation Modifications**
Please use this section for editorial suggestions as well as relatively minor modifications of existing data that would enhance clarity.

Reviewer #2: I am find with their reply on my minor issues.

Reviewer #3: I found some minor issues (see below) in the revised manuscript and would like to ask the authors to correct them.

Page 2, Line 31: “Ustilago maydis” should be italic.
Page 2, Line 34: “in planta” should be italic.
Page 3, Line 50: “pleiades” should be italic (only for p).
Page 7, Line 157: “SPtay1mCherry-Tay1” is better to be SPtay1-mCherry-Tay1, to be consistent with “SPmer1-mCherry-Mer1” from line 156. Same for page 27, Line 792.
Page 10, Line 257: For the description of high stringency media, I suggest not to use abbreviated letters like LWHA when it appears at first place in text.
Page 12, Line 300: Through the entire manuscript, the word “knockout” or “knock out” should be consistent (e.g. Line 304, “knockouts” and “knock out”).
Page 13, Line 330: “our results indicate, that” I don’t think a comma goes there.
Page 19, Line 501: “Einheitserde” I assume that this is commercial name. Could you provide information?
Page 28, Line 855: “U. maydis” should be italic.
Page 29, Line 861, 862: “SPMer1mCherry-Mer1” is better to be SPMer1-mCherry-Mer1.
Page 29, Line 875: “H202” should be “H2O2”.
Page 29, Line 899: There is period two times after “50µm”.
Figure 6: For broad readers, the schematic picture is hard to understand what it actually depicts. Please reconsider it and provide more professional figure and description in legend.

Answers:

We would like to thank the reviewers to read through the whole manuscript and to improve it further with their input. We have corrected all points mentioned. They can be found highlighted in the version with track changes.
Regarding the suggestion for Page 7, Line 157 we also changed “SP_mer1_-Mer1-3xHA” to “SP_mer1_-Mer1-mCherry-3xHA “which is the correct construct in this case.
We now use the phrase “knock out” throughout the text.
We also added information on the supplier of “Einheitserde”, a commercial horticultural substrate. Lines 501-502.
Following the advice of reviewer 3 we revised the model (Fig.6 showing now in more detail the proposed function of Mer1 and specifying fungal and plant cell. We also worked through and adapted the corresponding legend for enhanced clarity.

Once again, we thank all reviewers and the editor for taking the time and for all suggestions that lead to an improvement of the work.

Sincerely,

Fernando Navarrete and Armin Djamei
